# A novel method based on lesion expansion to assess plant disease severity

**DOI:** 10.3389/fpls.2025.1510663

**Published:** 2025-02-27

**Authors:** Feng Qin, Haiguang Wang, Qian Jiang, Hongli Wang

**Affiliations:** College of Plant Protection, China Agricultural University, Beijing, China

**Keywords:** plant disease, severity, disease assessment, image processing, lesion expansion, expansion coefficient, wheat stripe rust

## Abstract

**Introduction:**

Severity is a key indicator utilized in plant disease monitoring and pathogen-plant interaction phenotyping.

**Methods:**

A plant disease severity assessment method based on lesion expansion was proposed in this study to more accurately and quickly assess the severity of plant diseases for which the lesion area ratio of an investigated plant unit at each severity class in the corresponding severity grading standard is not the actual ratio of the lesion area to the area of the whole investigated plant unit. By taking wheat stripe rust caused by *Puccinia striiformis* f. sp. *tritici* as an example, after image segmentation operations of single diseased wheat leaves with wheat stripe rust, lesion expansion processing was carried out using nine method combinations of three proposed lesion expansion methods and three proposed lesion expansion coefficient determination methods, and then the severity assessments of single diseased wheat leaves were conducted.

**Results:**

The results showed that the accuracy of severity assessments of single diseased wheat leaves in each severity class was in the range of 78.00% to 100.00%. No matter which method was used to determine the lesion expansion coefficient/coefficients, the performance of the severity assessments of the single diseased leaves achieved after lesion expansion using lesion expansion method 3 (the lesion expansion method based on an image scaling algorithm) outperformed that achieved after lesion expansion using the other two lesion expansion methods. The performance of the method combination of lesion expansion method 3 and lesion expansion coefficient determination method 1 with a lesion expansion coefficient of 2.74, achieving an accuracy of 96.16% for severity assessments of all the single diseased wheat leaves, was the optimal method among the nine method combinations.

**Discussion:**

The results demonstrated that satisfactory severity assessment results could be achieved using the proposed method based on lesion expansion. The results indicated that the lesion-expansion-based plant disease severity assessment method is feasible, and can be used to solve the severity assessment problem described above. This study provided a new idea and method for accurate severity assessment of plant diseases and provided support for the automatic and intelligent assessment of plant disease severity.

## 1 Introduction

Plant diseases can reduce the yield and quality of plant products and affect food security worldwide. Some pathogens can produce various toxins when they infect host plants and cause diseases, resulting in serious harm to humans and livestock. Furthermore, some plant diseases can have serious impacts on ecological environments. Therefore, it is of great significance to strengthen the scientific management of plant diseases. Carrying out disease investigations and obtaining information on the occurrence of plant diseases is the basis and premise for the scientific and effective management of diseases. Severity is a key indicator used in plant disease investigations, plant disease monitoring, and diseased plant phenotyping, and the severity assessment is critical to understand the disease intensity, explore the pathogen-plant interaction, and implement disease management (Bock et al., 2022a; Jiang et al., 2022, 2023). The severity assessments of plant diseases mainly rely on the naked-eye observations (visual observations) of raters or assessors, but under laboratory conditions, the graph paper method and paper-weighing method can sometimes be used for disease severity assessments (Li et al., 2011). With the development of science and technology, the methods of plant disease investigation and monitoring supported by information technology have been developing rapidly (Wang, 2022, 2023). There are more and more applications of information technology in plant disease severity assessments, mainly including image processing technology (Li et al., 2011, 2013a; Barbedo, 2014; Pethybridge and Nelson, 2015; Shrivastava et al., 2015; Jiang et al., 2021; Olivoto et al., 2022; Yao et al., 2024), remote sensing technology (Huang et al., 2004; Wang et al., 2007, 2012, 2016; Zhao et al., 2014), and near-infrared spectroscopy technology (Li et al., 2015). In particular, image processing technology is more and more widely used in plant disease severity assessments (Li et al., 2011, 2013a; Barbedo, 2014; Pethybridge and Nelson, 2015; Shrivastava et al., 2015; Jiang et al., 2021; Olivoto et al., 2022; Yao et al., 2024). The applications of image processing technology, remote sensing technology, near-infrared spectroscopy technology, and other information technologies, provide strong support for more convenient and accurate severity assessments of plant diseases.

The severity assessments of plant diseases should be carried out in strict accordance with the severity grading standards of corresponding diseases. Severity is used to describe the disease intensity or infection degree on a plant unit (such as a leaf, fruit, stem, or plant), and in many plant disease severity grading standards, the severity classes are divided based on the ratios of the lesion areas to the areas of the investigated plant units (Nutter et al., 1991; Bock et al., 2022b). However, for some diseases, such as wheat stripe (yellow) rust caused by *Puccinia striiformis* f. sp. *tritici* (*Pst*) and wheat leaf rust caused by *P*. *triticina* (*Pt*), in their severity grading standards, the ratio of the lesion area to the area of an investigated plant unit corresponding to each severity class is not the actual ratio of the lesion area to the area of the investigated plant unit, which brings difficulties to the accurate severity assessments of these diseases. During the severity assessments of these diseases, it is easy to make errors in the assessment of the severity classes. For instance, according to the National Standard of the People’s Republic of China’s (GB/T 15795–2011) “Rules for Monitoring and Forecast of the Wheat Stripe Rust (*Puccinia striiformis* West.)”, based on the percentages of the lesion areas in the whole areas of the corresponding single diseased wheat leaves, eight severity classes including 1%, 5%, 10%, 20%, 40%, 60%, 80%, and 100% are classified for wheat stripe rust; if the percentage of the lesion area in the whole area of a single diseased wheat leaf infected with *Pst* is between two adjacent severity classes, the severity of this diseased wheat leaf is regarded as the nearest severity class; and if the percentage of the lesion area in the whole area of a single diseased wheat leaf infected with *Pst* is less than 1%, the severity of this diseased wheat leaf is recorded as the severity class of 1%. However, for each severity class of wheat stripe rust, the actual percentages of the lesion areas in the areas of the whole diseased wheat leaves are much lower than the lesion area percentage corresponding to the severity class in the severity grading standard of the disease, and even when the severity class of a single diseased wheat leaf infected with *Pst* is 100%, the diseased leaf is still not completely covered with the uredinia produced by *Pst*, and the actual percentage of the lesion area to the whole area of the single diseased leaf is much lower than 100% (Shang et al., 1990; Jiang et al., 2022).

The naked-eye observation method, namely the visual observation method, which is widely used in the severity assessments of plant diseases in practice, is time-consuming and laborious and is susceptible to the subjective experience of raters or assessors, and prone to resulting in assessment errors. To ensure the reliability of the assessment results, raters or assessors should be trained and familiar with the disease severity grading method before they can carry out the severity assessments using the naked-eye observation method. Both the graph paper and the paper-weighing methods are time-consuming, labor-intensive, complicated to carry out, and rarely used in production practice. The plant disease severity assessment methods based on remote sensing technology or near-infrared spectroscopy have a strong dependence on instruments with relatively high prices, thus, the methods are mainly used in research and still have a long way to go before being widely used in production practice. At present, when using image processing technology to assess the severity of a plant disease, a main research idea is to compare the actual ratio of the lesion area to the area of an investigated plant unit with the ratios of lesion areas to areas of the plant units corresponding to the severity classes in the severity grading standard of the disease and then to determine the severity class of the investigated plant unit (Li et al., 2011, 2013a; Pethybridge and Nelson, 2015; Shrivastava et al., 2015; Jiang et al., 2021; Olivoto et al., 2022; Yao et al., 2024). Thus, severity assessment errors can be induced for plant diseases with the severity grading standards in which the ratio of the lesion area to the area of an investigated plant unit corresponding to each severity class is not the actual ratio of the lesion area to the area of the investigated plant unit, exerting great effects on the application of the plant disease severity assessment method based on image processing technology. Therefore, it is of great significance to solve the problem caused by the inconsistency between the actual ratio of the lesion area to the area of an investigated plant unit and the lesion area ratio corresponding to the severity class in the plant disease severity grading standard, which is crucial to the accurate assessment of plant disease severity and is very important for the realization of automatic assessment of plant disease severity. Jiang et al. (2022) conducted image segmentation of wheat stripe rust and obtained the actual percentages of the lesion areas in the areas of the corresponding whole single diseased wheat leaves using image processing technology, and then proposed two severity assessment methods for wheat stripe rust based on the actual percentages of lesion areas, i.e., the severity assessment methods based on the midpoint-of-two-adjacent-means-based actual percentage reference range and the 90%, 95%, and 99% reference ranges of the actual percentages of lesion areas for each severity class, respectively. To realize the automatic severity assessments of wheat stripe rust based on the actual percentage of lesion areas, Jiang et al. (2024) built an automatic grading system of wheat stripe rust severity using an automatic image segmentation method and a severity assessment method based on the 99% reference range of the actual percentage of lesion areas for each severity class of wheat stripe rust. Furthermore, Jiang et al. (2023) proposed two severity assessment methods for wheat stripe rust based on the actual percentage of lesion areas using supervised learning and unsupervised learning, respectively. These methods proposed by Jiang et al. (2022, 2023, 2024) have provided good solutions to solve the problem described above.

In essence, the issue with the fact that the ratio of the lesion area to the area of an investigated plant unit corresponding to each severity class in the plant disease severity grading standard is not the actual ratio of the lesion area to the area of the investigated plant unit is the fact that the latter is much lower than the former. To better solve the problem regarding the inconsistency between the actual ratio of the lesion area to the area of an investigated plant unit and the lesion area ratio corresponding to the severity class in the plant disease severity grading standard, which can induce severity assessment errors, a severity assessment method based on lesion expansion for plant diseases was proposed in this study. In this study, wheat stripe rust was taken as the research object. In the images of each single diseased wheat leaf, using image processing technology, the segmentation of lesion/lesions was performed, the expansion of the segmented lesion/lesions was carried out to obtain new lesion/lesions, and then the area/pixel number of new lesion/lesions and the area/pixel number of the single diseased wheat leaf were calculated. Subsequently, the actual percentage of the lesion area in the area of the whole diseased wheat leaf was calculated. By comparing the actual percentage of the lesion area in the area of the whole diseased wheat leaf with the percentages in the eight severity classes in the disease severity grading standard, the severity of the single diseased wheat leaf was determined, finally. Using the proposed method, a severity assessment of single diseased wheat leaves with wheat stripe rust can be implemented. This study provides a novel method for the severity assessment of wheat stripe rust and can also provide a methodology and ideas for severity assessments of other similar plant diseases. Furthermore, a basis will be provided for the accurate and automatic severity assessment of plant diseases.

## 2 Materials and methods

### 2.1 Workflow for assessing plant disease severity based on lesion expansion

**Figure 1:**
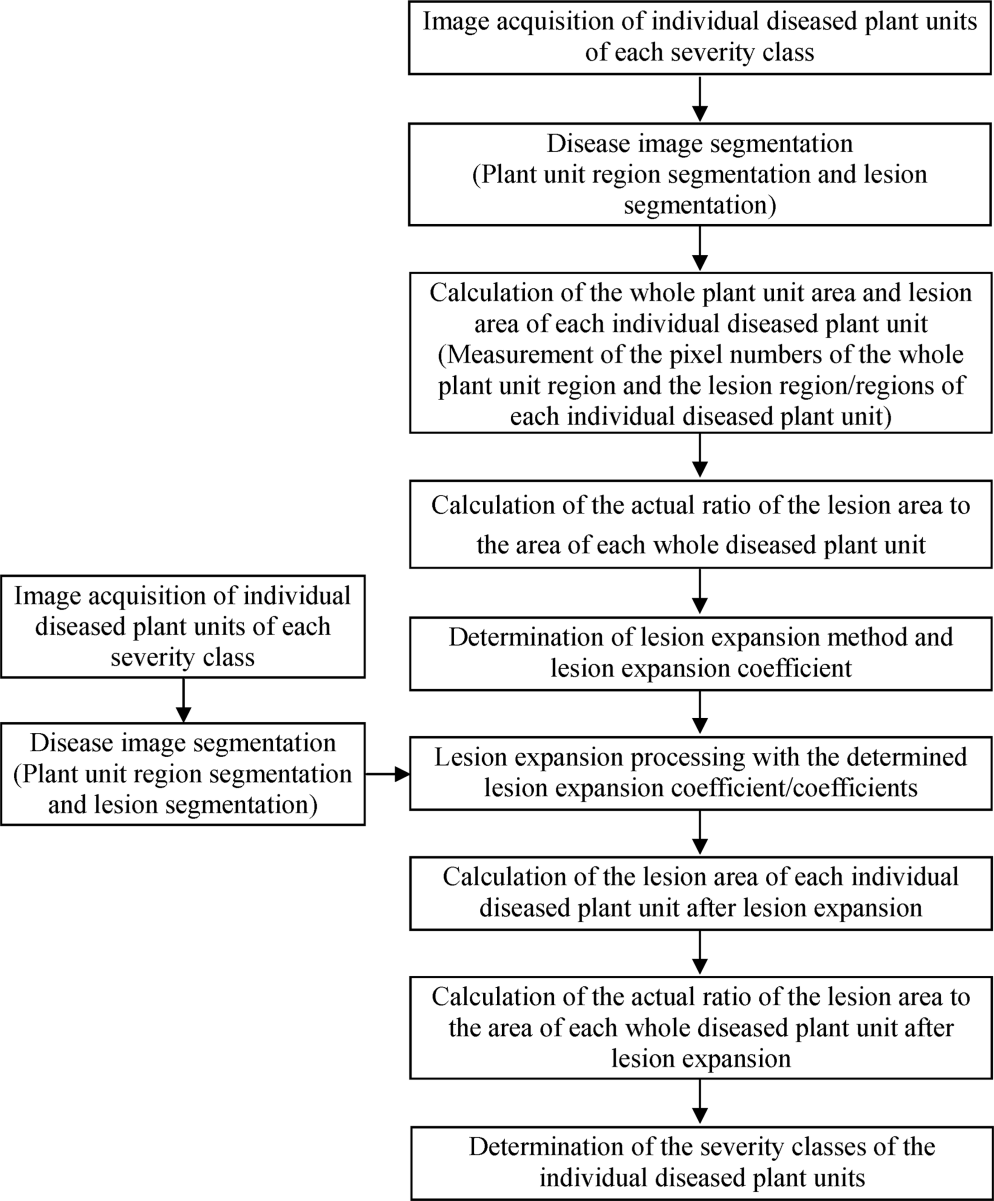
Workflow diagram for assessing plant disease severity based on lesion expansion.

In this study, a severity assessment method for plant diseases based on lesion expansion using image processing technology was proposed, and the workflow to implement the method is shown in **Figure 1**, mainly including the steps as follows.

1) Image acquisition of individual diseased plant units of each severity class

Various image acquisition devices such as digital cameras, mobile phones, or other devices with camera functions can be used to acquire images of individual diseased plant units of each severity class of plant disease.

2) Disease image segmentation

Image processing software is used to manually or automatically segment the diseased plant units from the backgrounds to obtain the plant unit region images, and then the segmented lesion images are obtained by segmenting the lesion region/lesion regions from the plant unit region images.

3) Calculation of the whole plant unit area and lesion area of each individual diseased plant unit

The area features of the plant unit region images and the corresponding segmented lesion images are extracted using image processing software, respectively, and then the whole plant unit area and lesion area of each individual diseased plant unit are obtained, or, to represent the areas by the pixel numbers, the number of the whole plant unit pixels and the number of the lesion pixels of each individual diseased plant unit are obtained by statistical calculation operations in image processing software.

4) Calculation of the actual ratio of the lesion area to the area of each whole diseased plant unit

Based on the obtained whole plant unit area and lesion area of each individual diseased plant unit, the actual ratio of the lesion area to the area of the corresponding whole diseased plant unit is calculated according to Formula (1), or, based on the obtained number of the whole plant unit pixels and number of the lesion pixels of each individual diseased plant unit, the actual ratio of the lesion area to the area of the corresponding whole diseased plant unit is calculated according to Formula (2).


(1) $$R_{\text{d}}=\frac{A_{\text{d}}}{A_{\text{u}}}\times 100\%$$

where *R*_d_ is the actual ratio of the lesion area to the area of the whole individual diseased plant unit, *A*_d_ is the lesion area in the whole individual diseased plant unit, and *A*_u_ is the area of the whole individual diseased plant unit.


(2) $$R_{\text{d}}=\frac{P_{\text{d}}}{P_{\text{u}}}\times 100\%$$

where *R*_d_ is the actual ratio of the lesion area to the area of the whole individual diseased plant unit, *P*_d_ is the pixel number of the lesion region/regions in the whole individual diseased plant unit, and *P*_u_ is the pixel number of the whole individual diseased plant unit.

5) Determination of lesion expansion method and lesion expansion coefficient

Based on the actual ratios of the lesion areas to the areas of the corresponding whole diseased plant units for all the severity classes of a disease, a variety of methods can be tried to determine the lesion expansion coefficients. For instance, the lesion area ratio in the severity grading standard corresponding to the severity class of a diseased plant unit is divided by the actual ratio of the lesion area to the area of the whole diseased plant unit, the calculation result is recorded as *r*, and then, the value of *r* corresponding to the diseased plant unit with the highest actual lesion area ratio among the diseased plant units with the highest severity class can be treated as the lesion expansion coefficient of the diseased plant units in all the severity classes. The value of *r* corresponding to the diseased plant unit with the highest actual lesion area ratio among the diseased plant units at a severity class can be treated as the lesion expansion coefficient of the diseased plant units in the corresponding severity class. The mean of the *r* values corresponding to the diseased plant units at a severity class can be treated as the lesion expansion coefficient of the diseased plant units in the corresponding severity class. The mean of the *r* values corresponding to the diseased plant units in all the severity classes can be treated as the lesion expansion coefficient of the diseased plant units at all the severity classes. Moreover, within a certain range, the enumeration method can be used to determine the lesion expansion coefficient of the diseased plant units in each severity class or the lesion expansion coefficient of the diseased plant units in all the severity classes.

6) Lesion expansion processing with the determined lesion expansion coefficient/coefficients

Using the lesion expansion coefficient/coefficients determined in the above step (Step 5), lesion expansion processing of each lesion image can be carried out based on the centroid of each lesion in image processing software. If the expansion range of a lesion exceeds the boundary of the diseased plant unit, the exceeded part should be removed to obtain the new expanded lesion.

7) Calculation of the lesion area of each individual diseased plant unit after lesion expansion

Using image processing software, the area features of the lesion images after lesion expansion are extracted, and then the lesion area of each individual diseased plant unit is obtained, or to represent the areas by the pixel numbers, the number of the lesion pixels of each individual diseased plant unit is obtained by statistical calculation operations in image processing software.

8) Calculation of the actual ratio of the lesion area to the area of each whole diseased plant unit after lesion expansion

Based on the obtained whole plant unit area and lesion area or the number of whole plant unit pixels and number of lesion pixels of each individual diseased plant unit after lesion expansion, the actual ratio of the lesion area to the area of the corresponding whole diseased plant unit after lesion expansion is calculated according to Formula (1) or (2) presented above.

9) Determination of the severity classes of the individual diseased plant units

By comparing the actual ratio of the lesion area to the area of each whole diseased plant unit after lesion expansion with the lesion area ratio for each severity class in the disease severity grading standard, the severity of the diseased plant unit is determined, or, efforts can be made to acquire disease image of an individual diseased plant unit, perform segmentation operations to obtain the plant unit region image and segmented lesion image, carry out lesion expansion based on the determined lesion expansion coefficient/coefficients, calculate the lesion area and the whole plant unit area of the individual diseased plant unit after lesion expansion, and calculate the actual ratio of the lesion area to the area of the whole diseased plant unit after lesion expansion, and, finally, the severity of the diseased plant unit can be determined by comparing the actual ratio of the lesion area to the area of the whole diseased plant unit after lesion expansion with the lesion area ratio for each severity class in the disease severity grading standard.

Hereinafter, wheat stripe rust will be taken as an example to illustrate how to implement the severity assessment of wheat stripe rust based on lesion expansion.

### 2.2 Disease image acquisition, disease image segmentation, and calculation of the actual percentage of the lesion area in the area of each whole single diseased wheat leaf

**Figure 2:**
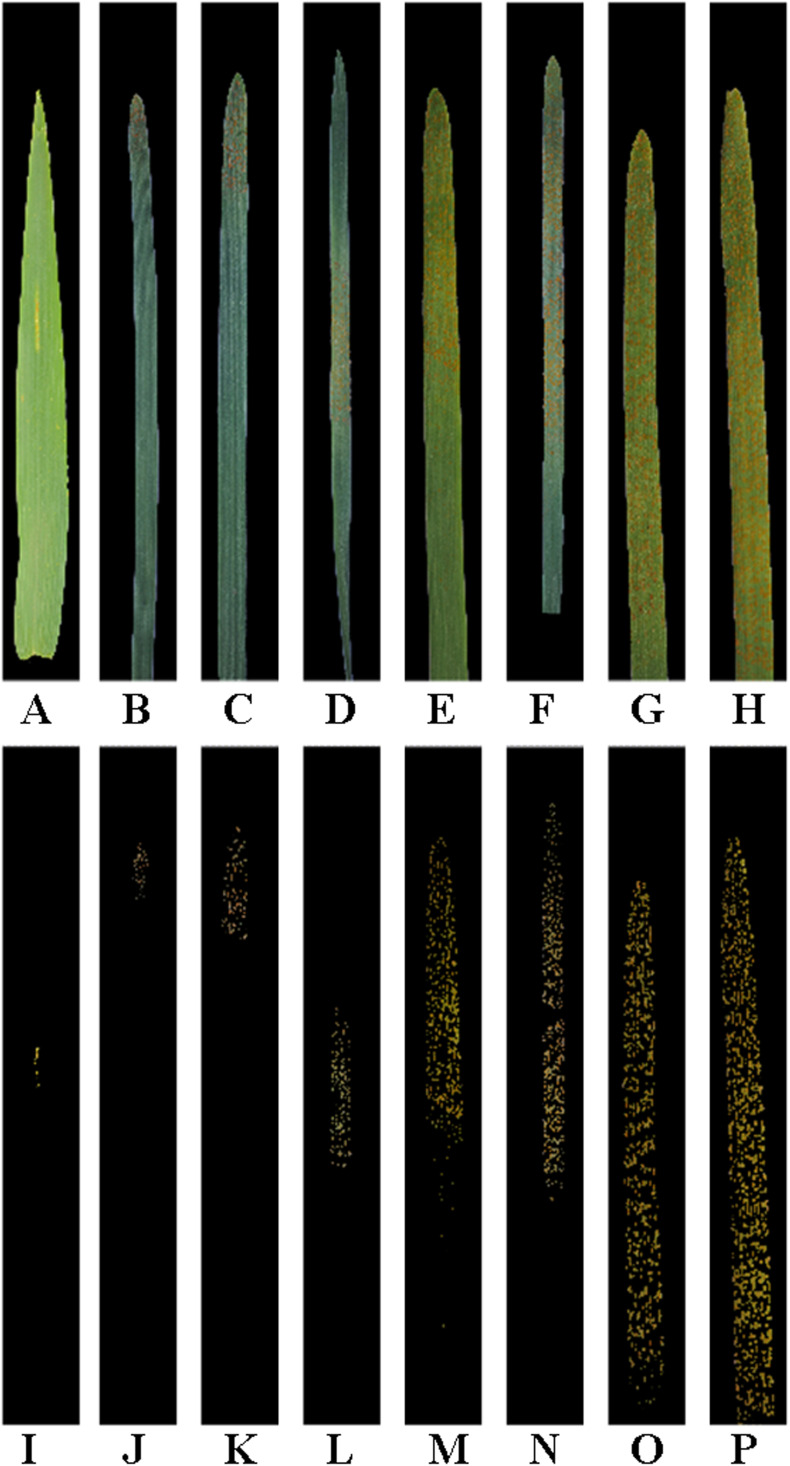
Leaf region images and lesion images after manual segmentation of the single wheat leaf images of each severity class of wheat stripe rust. All the images are shown after being cropped uniformly. **(A–H)** Original leaf region images after manual segmentation of the single diseased wheat leaf images in the severity classes of 1%, 5%, 10%, 20%, 40%, 60%, 80%, and 100%, respectively; **(I–P)** Original lesion images after manual segmentation of the single diseased wheat leaf images in the severity classes of 1%, 5%, 10%, 20%, 40%, 60%, 80%, and 100%, respectively.

In the previous study conducted by Jiang et al. (2022), after artificial spray inoculation of *Pst* on the wheat seedlings in the field and indoor environments, single diseased wheat leaves with typical symptoms at the severity classes of 1%, 5%, 10%, 20%, 40%, 60%, 80%, and 100% were collected according to the severity grading standard of wheat stripe rust (the National Standard of the People’s Republic of China (GB/T 15795–2011) as described above, and then an image of each single diseased wheat leaf was taken with a sheet of A4 white paper as background using a Nikon D700 digital camera (Nikon Corp., Tokyo, Japan), a HUAWEI P30 smartphone, or an iPhone 6S smartphone. For each severity class of wheat stripe rust, 50 images in the JPEG format (one image per single diseased wheat leaf) were acquired. A total of 400 images of the single diseased wheat leaves were acquired for the eight severity classes (i.e., 1%, 5%, 10%, 20%, 40%, 60%, 80%, and 100%). The resolutions of the acquired disease images were 4256×2832, 3648×2736, and 4032×3024 pixels, respectively, depending on the different devices used for disease image acquisition. Using the Adobe Photoshop 2022 software (Adobe Systems Incorporated, San Jose, CA, USA), the leaf region image and the lesion image, as shown in **Figure 2**, were manually segmented from each single diseased wheat leaf image, and then pixel statistics of the corresponding leaf region and lesion region/regions were implemented. Subsequently, the actual percentage of the lesion area in the area of each whole single diseased wheat leaf was achieved according to Formula (2) as described above.

### 2.3 Lesion expansion methods

A novel method based on lesion expansion for severity assessments of wheat stripe rust was proposed in this study because the actual percentage of the lesion area in the area of a whole single diseased wheat leaf infected with *Pst* is much lower than the lesion area percentage of the corresponding severity class in the severity grading standard of wheat stripe rust, as described above. The overall idea of this method was to conduct the expansion of the lesion/lesions in the image of a single diseased wheat leaf, obtain the area/pixel number of the new obtained lesion/lesions and the area/pixel number of the whole single diseased wheat leaf, calculate the actual percentage of the lesion area in the area of the whole single diseased wheat leaf according to Formula (1) or (2) as described above, and finally, compare the actual percentage of the lesion area in the area of the whole single diseased wheat leaf with the percentages for all the severity classes in the disease severity grading standard and achieve the severity class of the single diseased wheat leaf. According to the overall idea, in MATLAB 2019b software (MathWorks, Natick, MA, USA), the segmented lesion image of a whole single diseased wheat leaf infected with *Pst* was converted from the RGB image format to the grayscale image format using the rgb2gray function, and then the grayscale image was binarized using the Otsu method, with the lesion pixels as the foreground (the value of each lesion pixel was regarded as 1) and the remaining pixels in the single diseased wheat leaf image as the background (the value of each pixel in the non-lesion region of the single diseased wheat leaf image was regarded as 0). The connected component extraction algorithm was utilized to extract the connected component/components corresponding to the lesion/lesions from the binary image, and then the expansion of each lesion was carried out based on the corresponding centroid. The expanded lesion image was pasted back into the original single diseased wheat leaf image using centroid alignment, and if there were lesion pixels beyond the leaf region, the values of the corresponding pixels were reset to 0. Subsequently, the area/pixel number of the new obtained lesion/lesions after lesion expansion and the actual percentage of the corresponding lesion area in the area of the whole single diseased wheat leaf were calculated. Finally, the severity of the single diseased wheat leaf was assessed by comparing the actual percentage of the lesion area in the area of the whole single diseased wheat leaf with the percentages corresponding to all the eight severity classes in the disease severity grading standard of wheat stripe rust.

From the perspective of the specific implementation of lesion expansion, in this study, three methods to implement lesion expansion were proposed based on two ideas. The following are the two ideas in detail.

Idea 1. For each single diseased leaf image, based on the disease region/regions (namely, lesion/lesions; for wheat stripe rust, generally, a lesion region is actually the individual region covered with urediospores) in the segmented lesion image, the centroid of each disease region is located. Because the shapes of the lesions of many plant diseases are irregular, it is best to perform image processing based on the centroid of each lesion. Then, based on the distance from the centroid to each point on the boundary of the lesion region, the point extends outward (the line connecting the centroid to each point on the boundary of the lesion region extends outward in the direction from the centroid to the corresponding point on the lesion boundary), according to a determined lesion expansion coefficient. Subsequently, the extended ends are connected together to form a closed region (i.e., a new lesion). Under this idea, two methods for lesion boundary selection were proposed based on the external edge and the internal edge of the lesion, respectively, and thus two specific implementation methods of lesion expansion were developed, which were named the lesion expansion method based on the external edge of the lesion (i.e., lesion expansion method 1—the external edge-based lesion expansion method) and the lesion expansion method based on the internal edge of the lesion (i.e., lesion expansion method 2—the internal edge-based lesion expansion method), respectively.

Idea 2. For each single diseased leaf image, based on the disease region/regions (lesion/lesions) in the segmented lesion image, the centroid of each disease region is located. Because the shapes of the lesions of many plant diseases are irregular, it is best to perform image processing based on the centroid of each lesion. Then, based on the centroid, the lesion expansion is conducted based on a determined lesion expansion coefficient to form a new lesion (the whole lesion region expands outward from the centroid based on the determined lesion expansion coefficient, and thus a larger closed region is formed, which is a new lesion). Under this idea, the developed method for the specific implementation of lesion expansion was named the lesion expansion method based on the image scaling algorithm (i.e., lesion expansion method 3).

The three lesion expansion methods were specifically implemented as follows.

#### 2.3.1 Lesion expansion method 1—the external edge-based lesion expansion method

**Figure 3:**
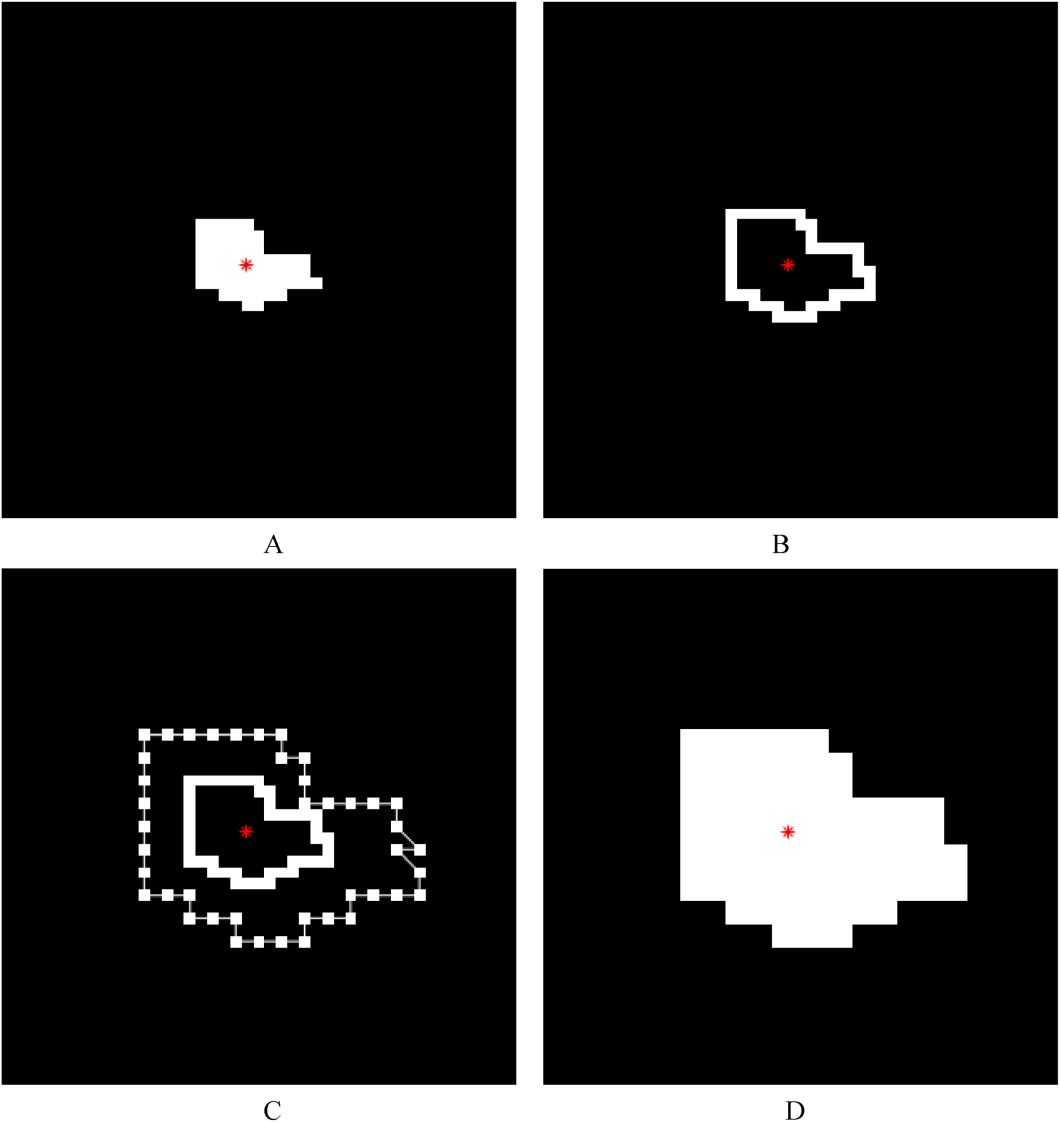
Illustrations of lesion image processing using lesion expansion method 1 (the external edge-based lesion expansion method). The red asterisk represents the centroid of a lesion. **(A)** The white region represents a lesion and the black region represents the background. **(B)** The external edge of each lesion (i.e., the boundary of each lesion region) was achieved after the original binary lesion image in **A** was subtracted from the image obtained by performing a morphological dilation operation on the lesion in **A** with a 3×3 structure element. **(C)** Based on the lesion expansion coefficient of 2 (*k*=2), the edge of the lesion was expanded outward, and then the expanded edge points were connected with straight lines in order. **(D)** The result (i.e., the new lesion obtained after lesion expansion) was achieved after the morphological hole-filling operation was performed on the connected region obtained by connecting the expanded edge points.

Based on the connected component/components corresponding to the lesion/lesions extracted from the binary image of the segmented lesion image of a whole single diseased leaf using the connected component extraction algorithm as described above, a morphological hole-filling operation was performed on the extracted connected component/components to remove the internal holes. The location of the centroid of each lesion was calculated. A morphological dilation operation was performed on the connected component/components with a structure element with the size of 3×3, and then the original binary lesion image was subtracted by the obtained image to achieve the edge of each lesion (i.e., the boundary of each lesion region). The external edge of each lesion was found in this way and ensured that the original lesion could be calculated when it was only a line or a point. Then, by taking the centroid of a lesion region as the center point, each edge point (i.e., each point on the boundary of the lesion region) was extended in the opposite direction of its connection with the centroid (that is, the direction away from the centroid). Assuming that the distance between an edge point and the centroid was *d* and the lesion expansion coefficient was set to *k*, the distance between the edge point after extension and the centroid was *d*×*k*. Subsequently, the ends of the edge points after extension were connected together to form a new expanded lesion, and the morphological hole-filling operation was performed on the new expanded lesion. The implementation of lesion expansion method 1 (the external edge-based lesion expansion method) is illustrated in **Figure 3**. Finally, the expanded lesion image was pasted back into the original single diseased leaf image using centroid alignment, and if there were lesion pixels beyond the leaf region, the values of the corresponding pixels were reset to 0. The color of the new obtained lesion after the expansion was filled with the average value of the *R*, *G*, and *B* components of the corresponding original lesion, and thus a single leaf image containing the new obtained lesion/lesions after the expansion was achieved.

#### 2.3.2 Lesion expansion method 2—the internal edge-based lesion expansion method

**Figure 4:**
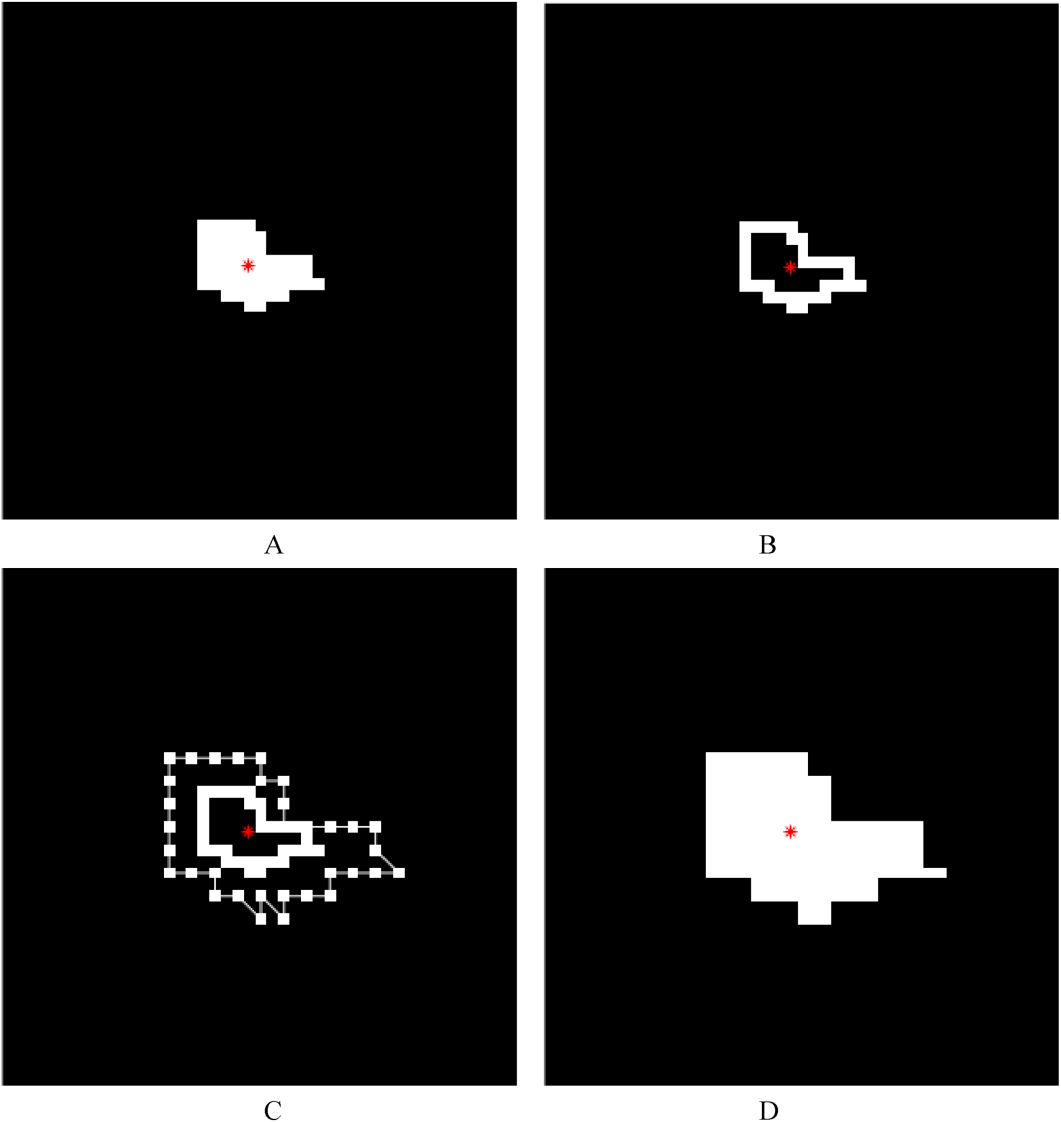
Illustrations of lesion image processing using lesion expansion method 2 (the internal edge-based lesion expansion method). The red asterisk represents the centroid of a lesion. **(A)** The white region represents a lesion and the black region represents the background. **(B)** The internal edge of each lesion (i.e., the boundary of each lesion region) was achieved after the image obtained by performing a morphological erosion operation on the lesion in **A** was subtracted from the original binary lesion image. **(C)** Based on the lesion expansion coefficient of 2 (*k*=2), the edge of the lesion was expanded outward, and then the expanded edge points were connected with straight lines in order. **(D)** The result (i.e., the new lesion obtained after lesion expansion) was achieved after the morphological hole-filling operation was performed on the connected region obtained by connecting the expanded edge points.

As in lesion expansion method 1 described above, based on the connected component/components corresponding to the lesion/lesions extracted from the binary image of the segmented lesion image of a whole single diseased leaf using the connected component extraction algorithm, first, a morphological hole-filling operation was performed on the extracted connected component/components to remove the internal holes. The centroid of each lesion was located and the corresponding location information was achieved. A morphological erosion operation was performed on the connected component/components and then the obtained image was subtracted by the original binary lesion image to achieve the edge of each lesion (i.e., the boundary of each lesion region). Thus, the internal edge of each lesion was found. If there was only one pixel in a lesion, then the pixel itself was the edge of the lesion, and in the case of a lesion with a single pixel and the case where the length or width of a lesion was one pixel, no expansion processing was performed. By taking the centroid of a lesion region as the center point, each edge point (i.e., each point on the boundary of the lesion region) was then extended in the opposite direction of its connection with the centroid (that is, the direction away from the centroid). Assuming that the distance between an edge point and the centroid was *d* and the lesion expansion coefficient was *k*, the distance between the edge point after extension and the centroid was *d*×*k*. Subsequently, the ends of the edge points after extension were connected together, and the morphological hole-filling operation was performed on the connected region to form a new expanded lesion. The implementation of lesion expansion method 2 (the internal edge-based lesion expansion method) is illustrated in **Figure 4**. The expanded lesion image was pasted back into the original single diseased leaf image using centroid alignment, and if there were lesion pixels beyond the leaf region, the values of the corresponding pixels were reset to 0. Finally, the color of the new obtained lesion after the expansion was filled with the average value of the *R*, *G*, and *B* components of the corresponding original lesion, and thus the single leaf image containing the new obtained lesion/lesions after the expansion was achieved.

#### 2.3.3 Lesion expansion method 3—the lesion expansion method based on an image scaling algorithm

**Figure 5:**
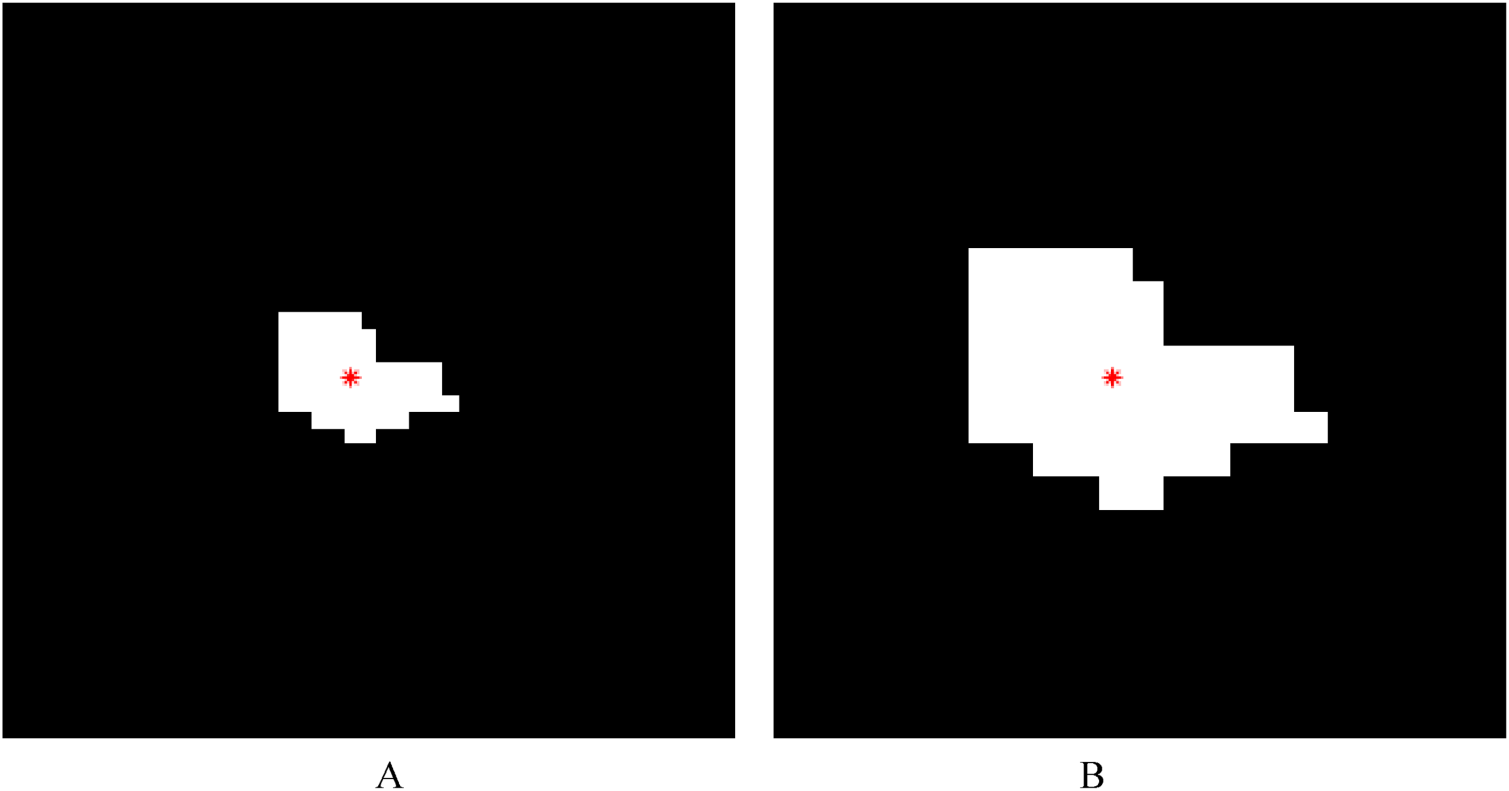
Illustrations of lesion image processing using lesion expansion method 3 (the lesion expansion method based on image scaling algorithm). The red asterisk represents the centroid of a lesion. **(A)** The white region represents a lesion and the black region represents the background. **(B)** Based on the centroid of the original binary lesion and a lesion expansion coefficient of 2 (*k*=2), the original binary lesion in **A** was enlarged. In the enlarged image, each non-zero pixel was considered as the lesion foreground and the corresponding value was set to 1, and each remaining pixel with the value of 0 was unchanged.

As in lesion expansion method 1 and lesion expansion method 2 described above, based on the connected component/components corresponding to the lesion/lesions extracted from the binary image of the segmented lesion image of a whole single diseased leaf using the connected component extraction algorithm, first, a morphological hole-filling operation was performed on the extracted connected component/components to remove the internal holes. The location of the centroid of each lesion was calculated and the corresponding location information was achieved. Given that the lesion expansion coefficient was set to *k*, the bicubic interpolation algorithm was used to enlarge each original binary lesion to *k* times based on the corresponding centroid. The enlarged image was then rebinarized, that is, each non-zero pixel was considered as the lesion foreground and the corresponding value was set to 1, and each pixel with the value of 0 remained unchanged. The implementation of lesion expansion method 3 (the lesion expansion method based on an image scaling algorithm) is illustrated in **Figure 5**. As described above, the expanded lesion image was pasted back into the original single diseased leaf image using centroid alignment, and if there were any lesion pixels beyond the leaf region, the values of the corresponding pixels were reset to 0. The color of the new obtained lesion after the expansion was filled with the average value of the *R*, *G*, and *B* components of the corresponding original lesion. Thus, a single leaf image containing the new obtained lesion/lesions after the expansion was achieved.

### 2.4 Methods to determine lesion expansion coefficients

The lesion expansion coefficient is crucial for lesion expansion processing, which has a great influence on the area of each expanded lesion, thus affecting the subsequent assessment of plant disease severity. In this study, the following three methods were used to determine the lesion expansion coefficients.

#### 2.4.1 Lesion expansion coefficient determination method 1

After the severity classes of the single diseased leaves were determined, the lesion area percentage in the severity grading standard corresponding to the severity class of a single diseased leaf was divided by the actual percentage of the lesion area to the area of the whole single diseased leaf. The calculation result was recorded as *r* and the value of *r* corresponding to the single diseased leaf with the highest actual lesion area percentage among the single diseased leaves with the highest severity class (100%) was recorded as Grate and was treated as the lesion expansion coefficient of the single diseased leaves in all the wheat stripe rust severity classes.

#### 2.4.2 Lesion expansion coefficient determination method 2

As in lesion expansion coefficient determination method 1 described above, after the severity classes of the single diseased leaves were determined, the lesion area percentage in the severity grading standard corresponding to the severity class of a single diseased leaf was divided by the actual percentage of the lesion area to the area of the whole single diseased leaf, and the calculation result was recorded as *r*. The value of *r* corresponding to the single diseased leaf with the highest actual lesion area percentage among the single diseased leaves in one of the severity classes was recorded as minrate*_S_* (in which, *S* was the severity class and it could be 1%, 5%, 10%, 20%, 40%, 60%, 80%, or 100%), which was treated as the lesion expansion coefficient of the single diseased leaves at the corresponding severity class (*S*). In particular, when the severity class was 100%, the value of minrate_100%_ was the same as the value of Grate obtained using lesion expansion coefficient determination method 1.

#### 2.4.3 Lesion expansion coefficient determination method 3

As in lesion expansion coefficient determination method 1 and lesion expansion coefficient determination method 2 described above, after the severity classes of the single diseased leaves were determined, the lesion area percentage in the severity grading standard corresponding to the severity class of a single diseased leaf was divided by the actual percentage of the lesion area to the area of the whole single diseased leaf, and the calculation result was recorded as *r*. The mean of the *r* values corresponding to the single diseased leaves in one of the severity classes was recorded as meanrate*_S_* (in which, *S* was the severity class and it could be 1%, 5%, 10%, 20%, 40%, 60%, 80%, or 100%), which was treated as the lesion expansion coefficient of the single diseased leaves at the corresponding severity class (*S*).

The lesion expansion coefficients for wheat stripe rust determined using the three lesion expansion coefficient determination methods described above are shown in **Table 1**. Based on the determined lesion expansion coefficients, lesion expansion processing of the lesion image of each single diseased leaf image was conducted using the lesion expansion methods described above. According to the processing results, the performances for severity assessments of wheat stripe rust using the three lesion expansion coefficient determination methods were compared. In fact, a total of nine specific method combinations for severity assessments were developed by combining the three lesion expansion methods with the three lesion expansion coefficient determination methods in this study.

**Table 1:** Lesion expansion coefficients for each severity class of wheat stripe rust determined using the three lesion expansion coefficient determination methods, respectively.

Lesion expansion coefficient determination method	Severity class	Lesion expansion coefficient
Lesion expansion coefficient determination method 1	All the severity classes of wheat stripe rust including 1%, 5%, 10%, 20%, 40%, 60%, 80%, and 100%	2.74 (Grate)
Lesion expansion coefficient determination method 2	1%	1.28 (minrate_1%_)
	5%	3.05 (minrate_5%_)
	10%	3.04 (minrate_10%_)
	20%	3.17 (minrate_20%_)
	40%	2.88 (minrate_40%_)
	60%	3.26 (minrate_60%_)
	80%	3.31 (minrate_80%_)
	100%	2.74 (minrate_100%_)
Lesion expansion coefficient determination method 3	1%	3.52 (meanrate_1%_)
	5%	4.06 (meanrate_5%_)
	10%	4.12 (meanrate_10%_)
	20%	4.17 (meanrate_20%_)
	40%	4.21 (meanrate_40%_)
	60%	3.63 (meanrate_60%_)
	80%	3.78 (meanrate_80%_)
	100%	3.31 (meanrate_100%_)

### 2.5 Calculation of the actual percentage of the lesion area in the area of each whole single diseased leaf after lesion expansion processing and severity assessment of the single diseased leaves

For each single diseased leaf containing the expanded lesion/lesions obtained by lesion image processing based on a determined lesion expansion coefficient method and a lesion expansion method, the number of the pixels of the whole single diseased leaf and the number of the lesion pixels after lesion expansion were calculated, respectively. The actual percentage of the lesion area in the area of the corresponding whole single diseased leaf after lesion expansion was then calculated according to Formula (2) presented above. Subsequently, the obtained actual lesion area percentage of each single diseased leaf was compared with the lesion area percentage for each severity class in the disease severity grading standard of wheat stripe rust as described above. Finally, the severity of each single diseased leaf was determined.

### 2.6 Evaluation of severity assessment performance

In this study, the accuracy of severity assessment, calculated using the maximum error reference method described by Xiao (1997), was utilized to evaluate severity assessment performance. The severity classes of wheat stripe rust of 1%, 5%, 10%, 20%, 40%, 60%, 80%, and 100% were represented by 1, 2, 3, 4, 5, 6, 7, and 8, respectively, and then the accuracy of the severity assessment of an individual single diseased leaf and the accuracy of severity assessments of multiple single diseased leaves were calculated using Formulas (3) and (4), respectively.


(3) $$s_{i}=(1-\frac{\left|{\hat{x}}_{i}-x_{i}\right|}{x_{i}\vee (x_{\text{max}}-x_{i})})\times 100\%$$

where *s_i_* is the accuracy of severity assessment of the *i*th individual single diseased leaf; ${\hat{x}}_{i}$ is the representative value of the severity class assessed for the *i*th individual single diseased leaf; *x_i_* is the representative value of the severity class determined by an actual survey for the *i*th individual single diseased leaf; *x*_max_ is the representative value of the highest severity class (100%) in the severity grading standard of wheat stripe rust, i.e., 8 in this study; and $x_{i}\vee (x_{\text{max}}-x_{i})$ is the maximum in *x_i_* and *x*_max_-*x_i_*, namely, the maximum error of the severity assessment of the *i*th individual single diseased leaf.


(4) $$s=\frac{1}{n}\sum_{i=1}^{n}(1-\frac{\left|{\hat{x}}_{i}-x_{i}\right|}{x_{i}\vee (x_{\text{max}}-x_{i})})\times 100\%$$

where *s* is the accuracy of severity assessments of a total of *n* single diseased leaves; *n* is the total number of the single diseased leaves assessed; ${\hat{x}}_{i}$ is the representative value of the severity class assessed for the *i*th individual single diseased leaf; *x_i_* is the representative value of the severity class determined by an actual survey for the *i*th individual single diseased leaf; *x*_max_ is the representative value of the highest severity class (100%) in the severity grading standard of wheat stripe rust, i.e., 8 in this study; and $x_{i}\vee (x_{\text{max}}-x_{i})$ is the maximum in *x_i_* and *x*_max_-*x_i_*, namely, the maximum error of the severity assessment of the *i*th individual single diseased leaf.

The accuracy of severity assessments of multiple single diseased leaves is the mean of the accuracies of severity assessments of multiple individual single diseased leaves. On the premise of obtaining the accuracies of severity assessments of multiple individual single diseased leaves, the accuracy of severity assessments of multiple single diseased leaves can be calculated using Formula (5).


(5) $$s=\frac{1}{n}\sum_{i=1}^{n}s_{i}$$

where *s* is the accuracy of severity assessments of a total of *n* single diseased leaves; *n* is the total number of the single diseased leaves assessed; *s_i_* is the accuracy of severity assessment of the *i*th individual single diseased leaf.

## 3 Results

**Figure 6:**
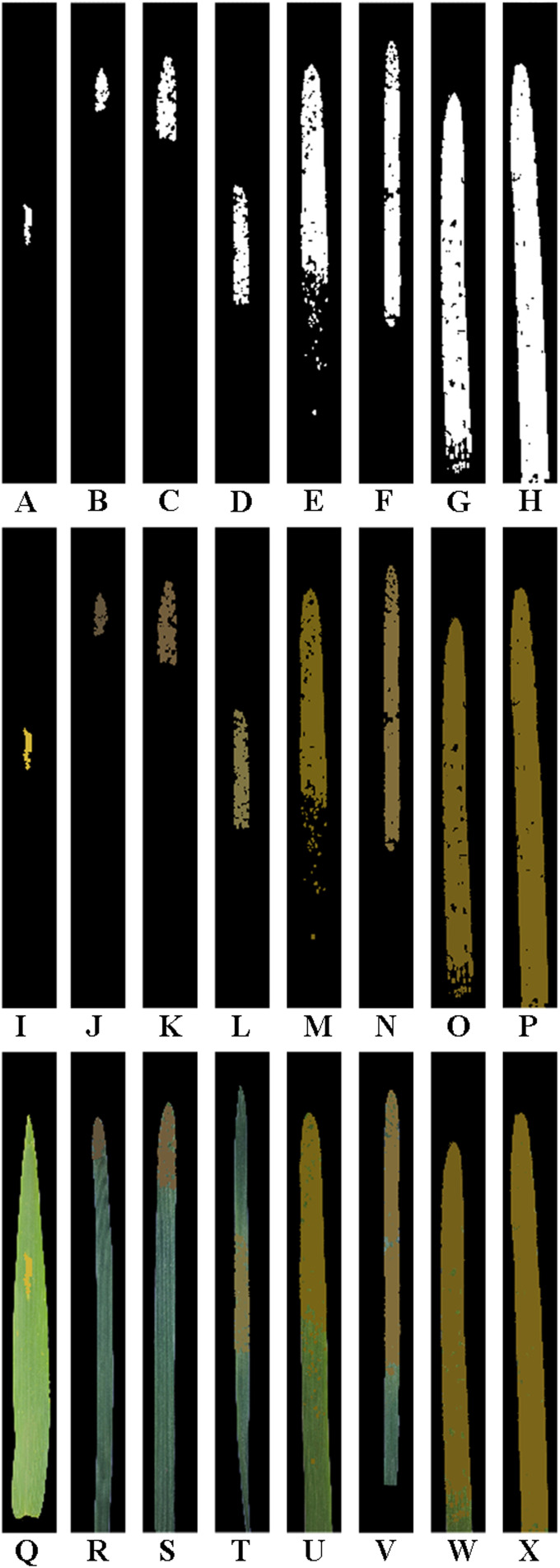
Results obtained after lesion expansion of the single wheat leaf images of each severity class of wheat stripe rust shown in **Figure 2** using lesion expansion method 1 (i.e., the external edge-based lesion expansion method) with a lesion expansion coefficient of 2.74 (i.e., Grate). To demonstrate more clearly, all the images are shown after being cropped uniformly. **(A–H)** The obtained images after lesion expansion of the segmented lesion images of the single diseased wheat leaf images in the severity classes of 1%, 5%, 10%, 20%, 40%, 60%, 80%, and 100%, respectively. **(I–P)** The obtained RGB images after lesion expansion of the segmented lesion images of the single diseased wheat leaf images in the severity classes of 1%, 5%, 10%, 20%, 40%, 60%, 80%, and 100%, respectively. **(Q–X)** The RGB images obtained after lesion expansion for the single diseased wheat leaves in the severity classes of 1%, 5%, 10%, 20%, 40%, 60%, 80%, and 100%, respectively.

**Figure 7:**
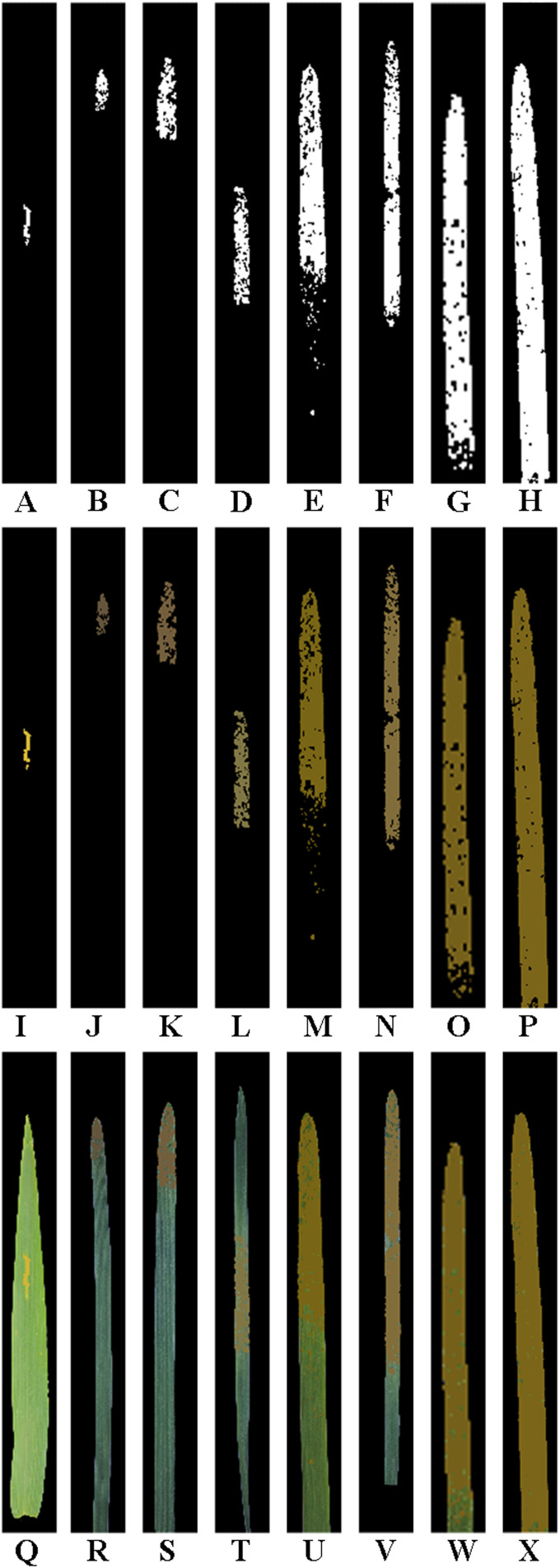
Results obtained after lesion expansion of the single wheat leaf images of each severity class of wheat stripe rust shown in **Figure 2** using lesion expansion method 2 (i.e., the internal edge-based lesion expansion method) with the lesion expansion coefficient of 2.74 (i.e., Grate). All the images are shown after being cropped uniformly. **(A–H)** The obtained images after lesion expansion of the segmented lesion images of the single diseased wheat leaf images in the severity classes of 1%, 5%, 10%, 20%, 40%, 60%, 80%, and 100%, respectively. **(I–P)** The obtained RGB images after lesion expansion of the segmented lesion images of the single diseased wheat leaf images in the severity classes of 1%, 5%, 10%, 20%, 40%, 60%, 80%, and 100%, respectively. **(Q–X)** The RGB images obtained after lesion expansion for the single diseased wheat leaves in the severity classes of 1%, 5%, 10%, 20%, 40%, 60%, 80%, and 100%, respectively.

**Figure 8:**
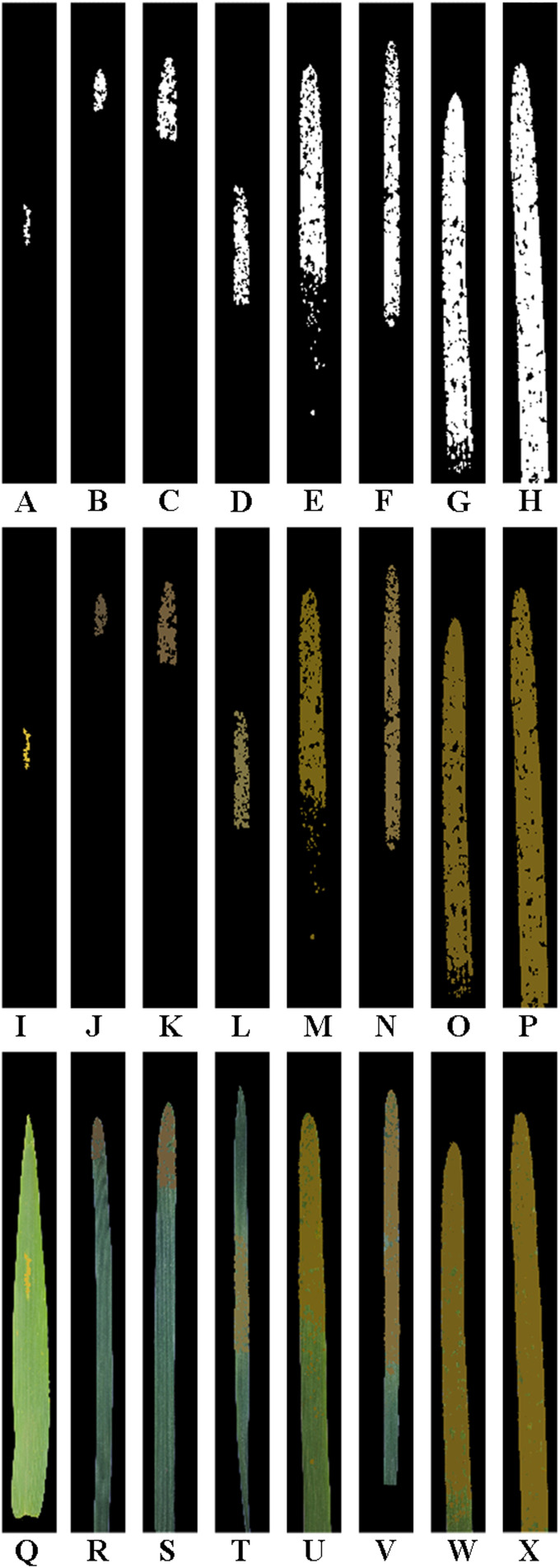
Results obtained after lesion expansion in the single wheat leaf images in each severity class of wheat stripe rust shown in **Figure 2** using lesion expansion method 3 (i.e., the lesion expansion method based on image scaling algorithm) with a lesion expansion coefficient of 2.74 (i.e., Grate). All the images are shown after being cropped uniformly. **(A–H)** The obtained images after lesion expansion of the segmented lesion images of the single diseased wheat leaf images in the severity classes of 1%, 5%, 10%, 20%, 40%, 60%, 80%, and 100%, respectively. **(I–P)** The obtained RGB images after lesion expansion of the segmented lesion images of the single diseased wheat leaf images in the severity classes of 1%, 5%, 10%, 20%, 40%, 60%, 80%, and 100%, respectively. **(Q–X)** The RGB images obtained after lesion expansion for the single diseased wheat leaves in the severity classes of 1%, 5%, 10%, 20%, 40%, 60%, 80%, and 100%, respectively.

Based on the lesion expansion coefficients determined using the three lesion expansion coefficient determination methods, the images of the single diseased wheat leaves in each severity class of wheat stripe rust were processed using the three lesion expansion methods described above, respectively. Severity assessments of the single diseased leaves were then performed based on the obtained actual percentages of the lesion areas after lesion expansion of the single diseased leaves in the area of the corresponding whole single diseased leaves, according to the severity grading standard of wheat stripe rust described above, and the results are shown in **Table 2**. The results (as shown in **Figures 6**–**8**) obtained after processing the images of the single diseased wheat leaves in each severity class of wheat stripe rust using the three lesion expansion methods with the lesion expansion coefficient of 2.74 (i.e., Grate), were taken as the examples to illustrate the results achieved after lesion expansion processing. In particular, the results achieved after lesion expansion processing using lesion expansion method 1 (i.e., the external edge-based lesion expansion method), lesion expansion method 2 (i.e., the internal edge-based lesion expansion method), and lesion expansion method 3 (i.e., the lesion expansion method based on image scaling algorithm) are shown in **Figure 6**, **Figure 7**, and **Figure 8**, respectively.

**Table 2:** Severity assessment results of the single diseased wheat leaves in each severity class of wheat stripe rust after lesion expansion processing using the three lesion expansion methods with the lesion expansion coefficients determined using the three lesion expansion coefficient determination methods, respectively.

Severity class	Representative value of severity class	Lesion expansion coefficient	Accuracy (*s*)		
			Lesion expansion method 1	Lesion expansion method 2	Lesion expansion method 3
1%	1	2.74 (Grate)	86.86%	88.00%	88.00%
1%	1	1.28 (minrate_1%_)	92.29%	97.14%	98.57%
1%	1	3.52 (meanrate_1%_)	85.43%	86.57%	86.57%
5%	2	2.74 (Grate)	87.33%	92.67%	95.00%
5%	2	3.05 (minrate_5%_)	85.67%	90.00%	89.33%
5%	2	4.06 (meanrate_5%_)	78.00%	82.33%	83.00%
10%	3	2.74 (Grate)	91.60%	99.20%	98.40%
10%	3	3.04 (minrate_10%_)	88.80%	94.80%	95.60%
10%	3	4.12 (meanrate_10%_)	82.80%	87.20%	85.60%
20%	4	2.74 (Grate)	94.50%	97.50%	99.00%
20%	4	3.17 (minrate_20%_)	91.00%	94.00%	95.50%
20%	4	4.17 (meanrate_20%_)	84.00%	88.00%	89.00%
40%	5	2.74 (Grate)	92.80%	95.60%	96.00%
40%	5	2.88 (minrate_40%_)	90.80%	94.00%	95.60%
40%	5	4.21 (meanrate_40%_)	80.00%	83.20%	85.20%
60%	6	2.74 (Grate)	92.33%	98.00%	98.67%
60%	6	3.26 (minrate_60%_)	90.00%	93.00%	93.67%
60%	6	3.63 (meanrate_60%_)	89.00%	91.33%	91.33%
80%	7	2.74 (Grate)	95.14%	98.86%	99.43%
80%	7	3.31 (minrate_80%_)	90.29%	95.14%	95.71%
80%	7	3.78 (meanrate_80%_)	89.14%	91.43%	92.00%
100%	8	2.74 (Grate/minrate_100%_)	100.00%	98.75%	94.75%
100%	8	3.31 (meanrate_100%_)	100.00%	99.75%	99.50%

In the table, each accuracy value is the mean of the accuracies of severity assessments of all the single diseased leaves at a severity class obtained using a combination method of a lesion expansion method and a lesion expansion coefficient determination method.

**Figure 9:**
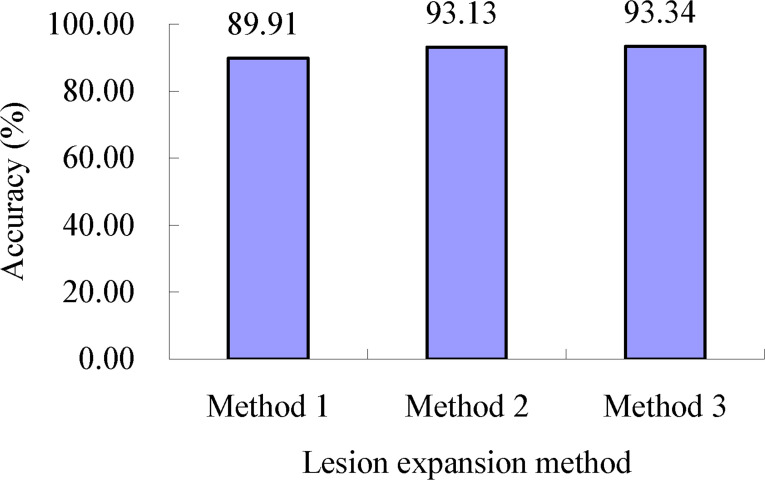
Severity assessment accuracies of all the single wheat leaves with wheat stripe rust when the lesion expansion operations were conducted under the condition without considering the lesion expansion coefficients using lesion expansion methods 1, 2, and 3, respectively. The value of 89.91% is the mean of the assessment accuracies of all the single wheat leaves infected with wheat stripe rust achieved when the lesion expansion operations were conducted using the three method combinations of lesion expansion method 1 and the three lesion expansion coefficient determination methods, respectively. The value of 93.13% is the mean of the assessment accuracies of all the single wheat leaves with wheat stripe rust achieved when the lesion expansion operations were conducted using the three method combinations of lesion expansion method 2 and the three lesion expansion coefficient determination methods, respectively. The value of 93.34% is the mean of the assessment accuracies of all the single wheat leaves with wheat stripe rust achieved when the lesion expansion operations were conducted using the three method combinations of lesion expansion method 3 and the three lesion expansion coefficient determination methods, respectively.

As shown in **Table 2**, after lesion expansion processing using lesion expansion method 1 with all the determined lesion expansion coefficients, respectively, the accuracy of severity assessments of the single diseased wheat leaves in each severity class of wheat stripe rust, namely, the mean of the accuracies of severity assessments of all the single diseased leaves in each severity class of wheat stripe rust, was in the range of 78.00% to 100.00%. After lesion expansion processing using lesion expansion method 2 with all the determined lesion expansion coefficients, respectively, the accuracy of severity assessments of the single diseased wheat leaves in each severity class of wheat stripe rust, namely, the mean of the accuracies of severity assessments of all the single diseased leaves in each severity class of wheat stripe rust, was in the range of 82.33% to 99.75%. After lesion expansion processing using lesion expansion method 3 with all the determined lesion expansion coefficients, respectively, the accuracy of severity assessments of the single diseased wheat leaves in each severity class of wheat stripe rust, namely, the mean of the accuracies of severity assessments of all the single diseased leaves in each severity class of wheat stripe rust, was in the range of 83.00% to 99.50%. When not considering the lesion expansion coefficients (namely, with all the lesion expansion coefficients determined using the three lesion expansion coefficient determination methods), the accuracies of severity assessments of all the single wheat leaves with wheat stripe rust when the lesion expansion operations were conducted using lesion expansion methods 1, 2, and 3, were 89.91%, 93.13%, and 93.34%, respectively, as shown in **Figure 9**. The results indicated that, overall, the best severity assessment performance for wheat stripe rust was achieved using lesion expansion method 3 to conduct lesion expansion, the performance of severity assessment of wheat stripe rust achieved using lesion expansion method 2 to conduct lesion expansion ranked second, and the severity assessment performance achieved using lesion expansion method 1 to conduct lesion expansion ranked third.

**Figure 10:**
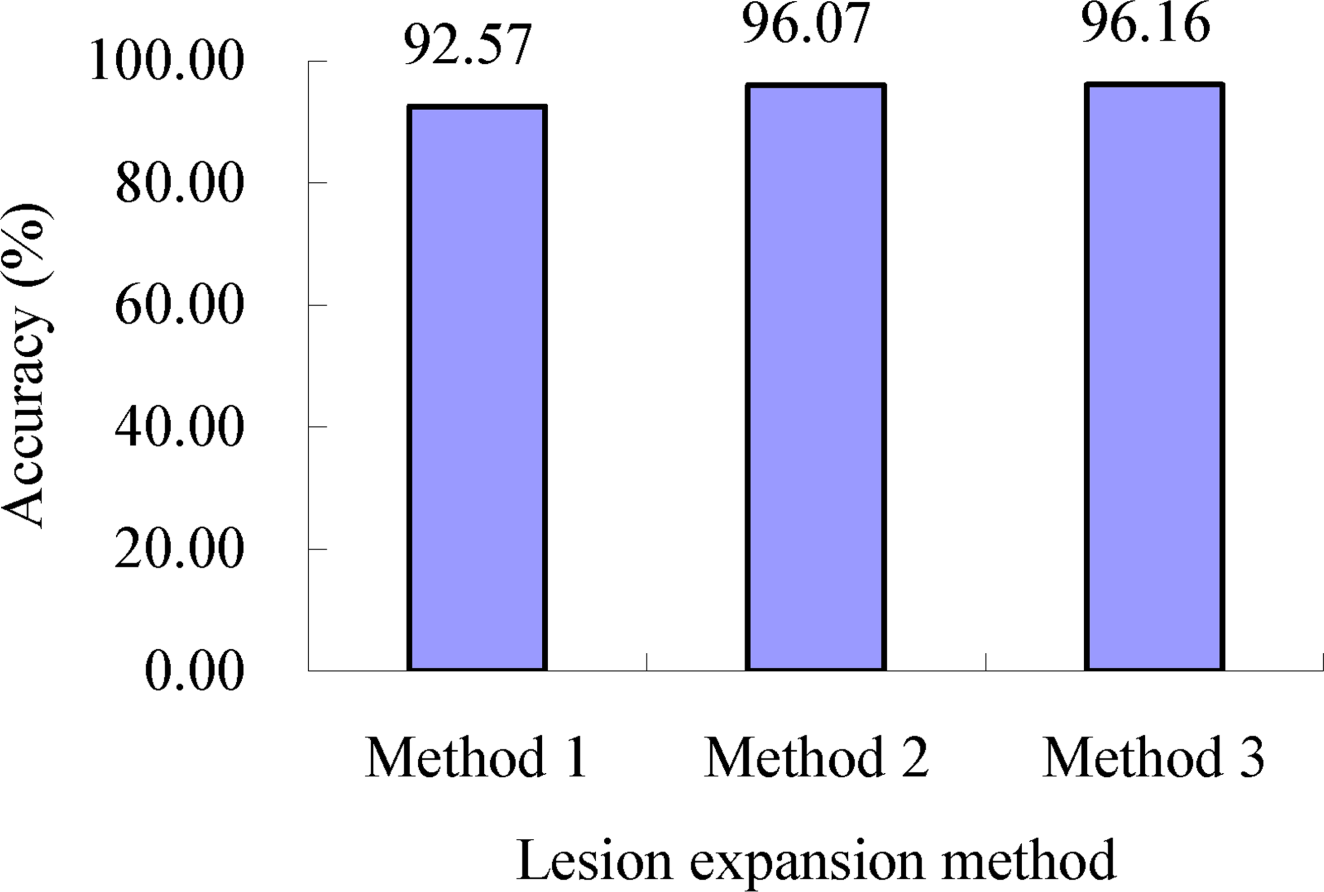
Severity assessment accuracies of all the single wheat leaves infected with wheat stripe rust when the lesion expansion operations were conducted with the lesion expansion coefficient of 2.74 (Grate) using lesion expansion methods 1, 2, and 3, respectively. The value of 92.57% is the mean of the assessment accuracies of all the single wheat leaves infected with wheat stripe rust achieved when the lesion expansion operations were conducted using the method combination of lesion expansion method 1 and lesion expansion coefficient determination method 1. The value of 96.07% is the mean of the assessment accuracies of all the single wheat leaves infected with wheat stripe rust achieved when the lesion expansion operations were conducted using the method combination of lesion expansion method 2 and lesion expansion coefficient determination method 1. The value of 96.16% is the mean of the assessment accuracies of all the single wheat leaves infected with wheat stripe rust achieved when the lesion expansion operations were conducted using the method combination of lesion expansion method 3 and lesion expansion coefficient determination method 1.

As shown in **Table 2**, for the lesion expansion coefficient of 2.74 (Grate) determined using lesion expansion coefficient determination method 1, when lesion expansion processing was conducted using lesion expansion method 1, the accuracy of the severity assessments of the single diseased wheat leaves in each severity class of wheat stripe rust, namely, the mean of the accuracies of severity assessments of all the single diseased leaves in each severity class of wheat stripe rust, was in the range of 86.68% to 100.00%, and the accuracy of severity assessments of all the single wheat leaves infected with wheat stripe rust was 92.57% (as shown in **Figure 10**). When lesion expansion processing was conducted using lesion expansion method 2, the accuracy of severity assessments of the single diseased wheat leaves in each severity class of wheat stripe rust, namely, the mean of the accuracies of severity assessments of all the single diseased leaves in each severity class of wheat stripe rust, was in the range of 88.00% to 99.20%, and the accuracy of severity assessments of all the single wheat leaves infected with wheat stripe rust was 96.07% (as shown in **Figure 10**). Finally, when lesion expansion processing was conducted using lesion expansion method 3, the accuracy of severity assessments of the single diseased wheat leaves in each severity class of wheat stripe rust, namely, the mean of the accuracies of severity assessments of all the single diseased leaves in each severity class of wheat stripe rust, was in the range of 88.00% to 99.43%, and the accuracy of severity assessments of all the single wheat leaves infected with wheat stripe rust was 96.16% (as shown in **Figure 10**). The results indicated that, overall, based on the lesion expansion coefficient of 2.74 (Grate) determined using lesion expansion coefficient determination method 1, the performance for severity assessments of wheat stripe rust achieved using lesion expansion method 3 to conduct lesion expansion was the best, the severity assessment performance achieved using lesion expansion method 2 to conduct lesion expansion ranked second, and the severity assessment performance achieved using lesion expansion method 1 to conduct lesion expansion ranked third.

**Figure 11:**
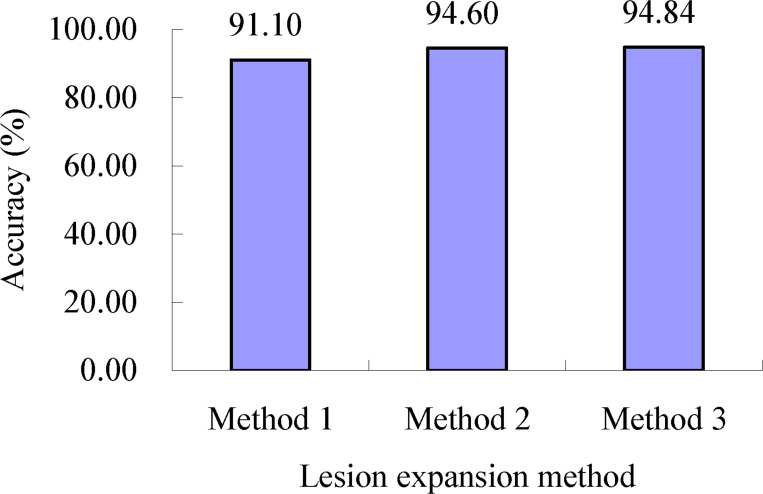
Severity assessment accuracies of all the single wheat leaves infected with wheat stripe rust when the lesion expansion operations were conducted with the lesion expansion coefficients minrate*_S_* using lesion expansion methods 1, 2, and 3, respectively. The value of 91.10% is the mean of the assessment accuracies of all the single wheat leaves infected with wheat stripe rust achieved when the lesion expansion operations were conducted using the method combination of lesion expansion method 1 and lesion expansion coefficient determination method 2. The value of 94.60% is the mean of the assessment accuracies of all the single wheat leaves infected with wheat stripe rust achieved when the lesion expansion operations were conducted using the method combination of lesion expansion method 2 and lesion expansion coefficient determination method 2. The value of 94.84% is the mean of the assessment accuracies of all the single wheat leaves infected with wheat stripe rust achieved when the lesion expansion operations were conducted using the method combination of lesion expansion method 3 and lesion expansion coefficient determination method 2.

As shown in **Table 2**, for the lesion expansion coefficients (minrate*_S_*) determined using lesion expansion coefficient determination method 2, when lesion expansion processing was conducted using lesion expansion method 1, the accuracy of severity assessments of the single diseased wheat leaves in each severity class of wheat stripe rust, namely, the mean of the accuracies of severity assessments of all the single diseased leaves in each severity class of wheat stripe rust, was in the range of 85.67% to 100.00%, and the accuracy of severity assessments of all the single wheat leaves infected with wheat stripe rust was 91.10% (as shown in **Figure 11**). When lesion expansion processing was conducted using lesion expansion method 2, the accuracy of severity assessments of the single diseased wheat leaves in each severity class of wheat stripe rust, namely, the mean of the accuracies of severity assessments of all the single diseased leaves in each severity class of wheat stripe rust, was in the range of 90.00% to 98.75%, and the accuracy of severity assessments of all the single wheat leaves infected with wheat stripe rust was 94.60% (as shown in **Figure 11**). When lesion expansion processing was conducted using lesion expansion method 3, the accuracy of severity assessments of the single diseased wheat leaves in each severity class of wheat stripe rust, namely, the mean of the accuracies of severity assessments of all the single diseased leaves in each severity class of wheat stripe rust, was in the range of 89.33% to 98.57%, and the accuracy of severity assessments of all the single wheat leaves infected with wheat stripe rust was 94.84% (as shown in **Figure 11**). The results indicated that, overall, based on the lesion expansion coefficients (minrate*_S_*) determined using lesion expansion coefficient determination method 2, the severity assessment performance for wheat stripe rust achieved when using lesion expansion method 3 to conduct lesion expansion was the best, and the severity assessment performances achieved when using lesion expansion method 2 and lesion expansion method 1 to conduct lesion expansion ranked second and third, respectively.

**Figure 12:**
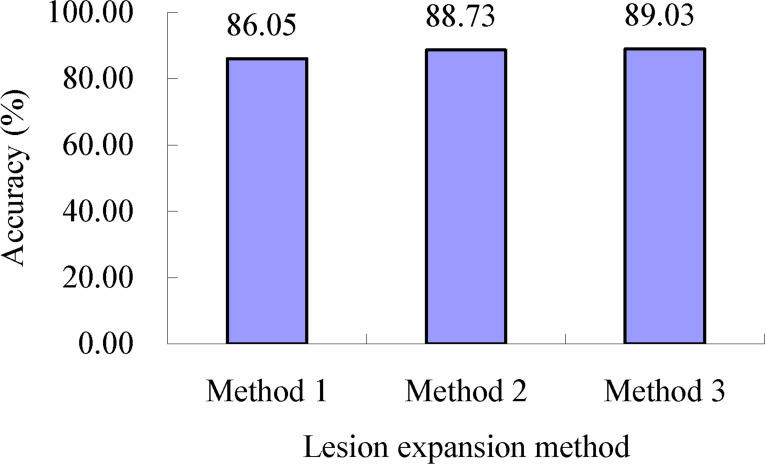
Severity assessment accuracies of all the single wheat leaves infected with wheat stripe rust when the lesion expansion operations were conducted with the lesion expansion coefficients meanrate*_S_* using lesion expansion methods 1, 2, and 3, respectively. The value of 86.05% is the mean of the assessment accuracies of all the single wheat leaves infected with wheat stripe rust achieved when the lesion expansion operations were conducted using the method combination of lesion expansion method 1 and lesion expansion coefficient determination method 3. The value of 88.73% is the mean of the assessment accuracies of all the single wheat leaves infected with wheat stripe rust achieved when the lesion expansion operations were conducted using the method combination of lesion expansion method 2 and lesion expansion coefficient determination method 3. The value of 89.03% is the mean of the assessment accuracies of all the single wheat leaves infected with wheat stripe rust achieved when the lesion expansion operations were conducted using the method combination of lesion expansion method 3 and lesion expansion coefficient determination method 3.

As shown in **Table 2**, for the lesion expansion coefficients (meanrate*_S_*) determined using lesion expansion coefficient determination method 3, when lesion expansion processing was conducted using lesion expansion method 1, the accuracy of severity assessments of the single diseased wheat leaves in each severity class of wheat stripe rust, namely, the mean of the accuracies of severity assessments of all the single diseased leaves in each severity class of wheat stripe rust, was in the range of 78.00% to 100.00%, and the accuracy of severity assessments of all the single wheat leaves infected with wheat stripe rust was 86.05% (as shown in **Figure 12**). When lesion expansion processing was conducted using lesion expansion method 2, the accuracy of severity assessments of the single diseased wheat leaves in each severity class of wheat stripe rust, namely, the mean of the accuracies of severity assessments of all the single diseased leaves in each severity class of wheat stripe rust, was in the range of 82.33% to 99.75%, and the accuracy of severity assessments of all the single wheat leaves infected with wheat stripe rust was 88.73% (as shown in **Figure 12**). When lesion expansion processing was conducted using lesion expansion method 3, the accuracy of severity assessments of the single diseased wheat leaves in each severity class of wheat stripe rust, namely, the mean of the accuracies of severity assessments of all the single diseased leaves in each severity class of wheat stripe rust, was in the range of 83.00% to 99.50%, and the accuracy of severity assessments of all the single wheat leaves infected with wheat stripe rust was 89.03% (as shown in **Figure 12**). The results indicated that, overall, based on the lesion expansion coefficients (meanrate*_S_*) determined using lesion expansion coefficient determination method 3, the best performance for severity assessments of wheat stripe rust was achieved using lesion expansion method 3 to conduct lesion expansion, and the severity assessment performances achieved using lesion expansion methods 2 and 1 to conduct lesion expansion ranked second and third, respectively.

The above results demonstrated that the satisfactory severity assessment results of the single diseased wheat leaves infected with *Pst* could be achieved using the proposed lesion expansion methods based on lesion expansion in this study. No matter which method was chosen from the three proposed lesion expansion coefficient determination methods to determine the lesion expansion coefficient/coefficients, satisfactory severity assessment performance for wheat stripe rust could be achieved when the three proposed lesion expansion methods were used for lesion expansion processing, respectively. Among the three lesion expansion methods, the best severity assessment performances were achieved using lesion expansion method 3 to conduct lesion expansion. Among the nine method combinations of the three lesion expansion methods and the three lesion expansion coefficient determination methods, the best severity assessment performance was achieved using the combination of lesion expansion method 3 and lesion expansion coefficient determination method 1. The results indicated that the proposed lesion expansion methods based on lesion expansion in this study can be used for severity assessments of single diseased leaves infected with wheat stripe rust.

## 4 Discussion

The plant disease severity assessment method proposed in this study is suitable for conducting severity assessments of any plant disease for which the actual ratio of the lesion area to the area of an investigated plant unit is much lower than the lesion area ratio corresponding to the determined severity class in the plant disease severity grading standard. By using the severity assessment method proposed in this study, based on image processing technology, the lesion expansion coefficient/coefficients can be easily obtained, and the new expanded lesion/lesions can also be easily obtained after lesion expansion processing. In practical applications, the appropriate lesion expansion coefficient/coefficients can be selected and the appropriate lesion expansion method can be used to conduct lesion expansion of the image of a diseased plant unit, and then the area of the new obtained lesion/lesions and the area of the whole diseased plant unit can be calculated. Subsequently, the actual percentage of the lesion area in the area of the whole diseased plant unit can be calculated, and the severity of the diseased plant unit can be determined according to the corresponding severity grading standard. Therefore, once the lesion expansion coefficient/coefficients and the lesion expansion method are determined, the accurate and rapid severity assessments of the diseased plant units can be conveniently realized, and the efficiency and performance of the severity assessments can be improved, which is of great significance for the surveying, monitoring, prediction, and control of plant diseases. Moreover, the methods proposed in this study can be used to expand the lesion/lesions in the image of a diseased plant unit to make the ratio of the area of the expanded lesion/lesions to the area of the investigated plant unit reach the lesion area ratio corresponding to the severity class in the severity grading standard as far as possible. The related image processing operations can be automatically performed using image processing technology. This study provided a method and idea for severity assessments of plant diseases, which is conducive to the realization of accurate and automatic assessments of plant disease severity.

In this study, based on the fact that the actual ratio of the lesion area to the area of an investigated plant unit is much lower than the lesion area ratio corresponding to the determined severity class in the plant disease severity grading standard, a novel severity assessment method of the diseased plant units of the corresponding plant diseases was proposed. The proposed method is conducive to the accurate assessment of the severity of plant diseases and the implementation of automated and intelligent severity assessment of plant diseases. The results obtained in this study indicated that it is feasible to conduct severity assessments of plant diseases after lesion expansion processing and calculating the ratios of the lesion areas to the areas of the corresponding diseased plant units and that the key point is to select the appropriate lesion expansion method and the appropriate lesion expansion coefficient/coefficients. According to the methods provided in this study, the appropriate lesion expansion method and the appropriate lesion expansion coefficient/coefficients can be determined and selected for severity assessments of diseased plant units.

In this study, after lesion expansion processing using the method combinations of the three lesion expansion coefficient determination methods and the three lesion expansion methods, severity assessments of single diseased wheat leaves infected with *Pst* were conducted, and satisfactory severity assessment performance for wheat stripe rust was achieved. In the practical application of the methods proposed in this study for plant disease severity assessments, for convenience, lesion expansion coefficient determination method 1 can be used to determine the lesion expansion coefficient for lesion expansion processing, because the performance of the lesion expansion coefficient determined using this method was the best among the lesion expansion coefficients determined using the three lesion expansion coefficient determination methods. The results obtained in this study indicated that the severity assessment performance decreased with an increase in the lesion expansion coefficient. Because the mean of the *r* values corresponding to the single diseased wheat leaves in all the severity classes was greater than the value of Grate of 2.74, in this study, no further attempt was made to use the mean of the *r* values corresponding to the single diseased wheat leaves in all the severity classes as the lesion expansion coefficient. In further studies, the lesion expansion coefficient for the single diseased leaves in each individual severity class can be set in a certain range, and then, based on the values obtained using the enumeration method with a searching step, lesion expansion processing operations can be performed on the images of the single diseased leaves in the severity class using the lesion expansion methods described above. Subsequently, according to the processing results, the lesion expansion coefficient with the best severity assessment performance can be treated as the lesion expansion coefficient of the corresponding severity class, thus achieving a lesion expansion coefficient for each severity class. In another way, the lesion expansion coefficient can be set in a certain range, and then, based on the values obtained using the enumeration method with a searching step, lesion expansion processing operations can be performed on the images of the single diseased leaves in all the severity classes using the lesion expansion methods described above. Subsequently, the lesion expansion coefficient with the best severity assessment performance can be treated as the lesion expansion coefficient for all the severity classes. In addition, the functional relationship between *r* and the actual lesion area percentage can be established based on machine learning, and then, based on the actual lesion area percentage of each single diseased leaf, the corresponding *r* value can be calculated and can subsequently be used as the lesion expansion coefficient of the corresponding single diseased leaf.

For plant diseases in which the severity classes are divided based on the ratios of the lesion areas to the areas of the investigated plant units, the lesion expansion coefficients should be greater than or equal to 1. When the lesion area ratios corresponding to the severity classes in the severity grading standards of the plant diseases are the actual lesion area ratios, the lesion expansion coefficients can be considered to be equal to 1, and the severity classes of the diseased plant units can be determined by directly comparing the actual lesion area ratios with the lesion area ratios corresponding to the severity classes in the severity grading standards, without requiring any lesion expansion operations. When the lesion area ratios corresponding to the severity classes in the severity grading standards of the plant diseases are not the actual lesion area ratios, the lesion expansion coefficients should be greater than 1, and the disease severity assessments can be conducted according to the lesion expansion-based methods proposed in this study. The maximum values of the lesion expansion coefficients may be different in specific plant diseases.

Disease image identification technology is developing rapidly and is increasingly widely applied in plant disease surveys and monitoring. There are many applications of deep learning technology, which has become the mainstream in plant disease image identification. However, traditional image identification technology also has its own advantages, especially when the number of plant disease images is not enough. Image segmentation has always been a hotspot and challenge in image processing research, and if the target/targets can be accurately segmented from the images, it will lay a very good foundation for target identification. In recent years, deep learning has been applied in the field of plant disease image segmentation (Zhang et al., 2020; Li et al., 2022). In future studies, image segmentation methods based on deep learning can be used to carry out the lesion image segmentation, and then the number of the segmented lesion pixels and the number of pixels in the corresponding single diseased leaf or the area of segmented lesion/lesions and the area of the corresponding single diseased leaf can be obtained. Subsequently, the severity of the corresponding single diseased leaf can be assessed, according to the corresponding severity grading standard.

The determination of the lesion boundary/boundaries in the image of a single diseased leaf is a very important step in the severity assessment of the single diseased leaf using the severity assessment methods proposed in this study. When the boundary of a lesion is determined, the distances between the centroid of the lesion and the points on the boundary of the lesion can be determined, and then, based on the distances and the determined lesion expansion coefficient, the lesion can be expanded outward. In this study, two methods were used to determine the lesion boundary/boundaries, and thus two lesion expansion methods (i.e., lesion expansion method 1—the external edge-based lesion expansion method and lesion expansion method 2—the internal edge-based lesion expansion method) were developed. The results obtained in this study indicated that satisfactory severity assessment performance can be achieved using the severity assessment methods based on the two lesion expansion methods. The severity assessment method based on lesion expansion method 2 outperformed that based on lesion expansion method 1, i.e., the severity assessment performance obtained after lesion expansion processing using the internal edge-based lesion expansion method was better than that obtained after lesion expansion processing using the external edge-based lesion expansion method.

When using a lesion expansion method to process the lesion images of plant diseases, there may be situations in which the expansion range of a lesion may exceed the boundary of the diseased plant unit, and the expanded lesions are connected together. After lesion expansion, the expanded lesion region/regions cannot exceed the boundary of the corresponding diseased plant unit. However, in practice, after a lesion is processed using a lesion expansion method, the new expanded lesion may exceed the boundary of the corresponding diseased plant unit, especially for the lesions that are originally located at or near the edge of the corresponding diseased plant unit. Therefore, the region range of the new expanded lesion should be limited based on the segmented plant unit image obtained before lesion expansion processing and the new lesion image obtained after lesion expansion processing, thus avoiding the region/regions exceeding the boundary of the plant unit that may be included into the new expanded lesion region when calculating lesion area. After lesion expansion processing, the adjacent lesions may be connected together. For the lesions connected together after lesion expansion, methods such as the watershed segmentation method (Li et al., 2013b) can be used to separate the connected lesions. If the connected lesions cannot be separated, multiple lesions connected together can be treated as one lesion.

There are two main ideas based on image processing technology to conduct plant disease severity assessments (Jiang et al., 2022, 2023, 2024). One idea, as described above, is to obtain the actual ratio of the lesion area to the area of an investigated diseased plant unit and then to compare the actual lesion area ratio with the lesion area ratios corresponding to all the severity classes in the disease severity grading standard to determine the severity class of the investigated diseased plant unit (Li et al., 2011, 2013a; Pethybridge and Nelson, 2015; Shrivastava et al., 2015; Jiang et al., 2021, 2022, 2023, 2024; Olivoto et al., 2022; Yao et al., 2024). The other idea is to obtain the features of the investigated diseased plant units and then build a plant disease severity assessment model based on the obtained features to determine the severity classes of the diseased plant units to be assessed (Bao et al., 2021; Jiang et al., 2022, 2023, 2024). However, when using the former, it is easy to make incorrect severity assessments for the plant diseases with the severity grading standards in which the lesion area ratios corresponding to all the severity classes are not the actual lesion area ratios (Jiang et al., 2022, 2023, 2024). Therefore, to solve this problem, based on the actual lesion area ratios, Jiang et al. (2022, 2023) proposed four severity assessment methods as described above, and a novel method was proposed in this study. In particular, for wheat stripe rust, satisfactory severity assessment results were achieved using the methods proposed by Jiang et al. (2022, 2023) and the novel lesion expansion-based methods proposed in this study, although some parameters used in these methods can be further optimized to obtain better severity assessment results. Rapidly developing deep learning technology can be used to build plant disease severity assessment models with the severity classes as different categories, and it is necessary to explore appropriate deep learning models for application. These methods can be used to develop severity assessment systems based on personal computers, Internet, and mobile terminals to facilitate plant disease severity assessments in practice.

## 5 Conclusion

In this study, a novel method based on lesion expansion to assess plant disease severity was proposed, and then, by taking wheat stripe rust as an example, the lesion expansion-based severity assessment methods for wheat stripe rust were proposed by combining three lesion expansion methods with three lesion expansion coefficient determination methods. Based on image processing technology, after lesion expansion processing of the single diseased wheat leaves using the nine method combinations of the three lesion expansion methods and the three lesion expansion coefficient determination methods, the area of each whole single diseased leaf and the area of the corresponding new obtained lesion/lesions after lesion expansion were calculated, respectively, and then, the actual percentage of the lesion area in the area of the corresponding whole single diseased leaf after lesion expansion was calculated. Subsequently, the obtained actual lesion area percentage of each single diseased leaf was compared with the lesion area percentage for each severity class in the disease severity grading standard of wheat stripe rust, and finally, the severity of each single diseased leaf was determined. The results showed that the accuracy of severity assessments of the single diseased wheat leaves in each severity class of wheat stripe rust (namely, the mean of the accuracies of severity assessments of all the single diseased leaves in each severity class of wheat stripe rust) was in the range of 78.00% to 100.00%, indicating satisfactory performance for severity assessments of the single diseased leaves infected with wheat stripe rust were achieved using the proposed methods based on lesion expansion. No matter which lesion expansion coefficient determination method was used, the severity assessment performance for the single diseased leaves infected with wheat stripe rust achieved using lesion expansion method 3 to conduct lesion expansion was the best, among the three lesion expansion methods. An accuracy of 96.16% for severity assessments of all the single wheat leaves infected with wheat stripe rust was achieved using the method combination of lesion expansion method 3 and lesion expansion coefficient determination method 1, indicating that this method combination is optimal among the nine method combinations of the three lesion expansion methods and the three lesion expansion coefficient determination methods. The results demonstrated that it is feasible to assess the severity of single diseased leaves infected with wheat stripe rust using the lesion-expansion-based severity assessment methods proposed in this study. This study provided a feasible solution to solve the problem of severity assessments for all the plant diseases in which the actual ratio of the lesion area to the area of an investigated plant unit is much lower than the lesion area ratio corresponding to the determined severity class in the plant disease severity grading standards, and provided a methodological reference for the accurate assessment of plant diseases.

## Data Availability

The original contributions presented in the study are included in the article/supplementary material. Further inquiries can be directed to the corresponding author.
